# How Do Viruses Avoid Inhibition by Endogenous Cellular MicroRNAs?

**DOI:** 10.1371/journal.ppat.1003694

**Published:** 2013-11-07

**Authors:** Bryan R. Cullen

**Affiliations:** Department of Molecular Genetics & Microbiology and Center for Virology, Duke University Medical Center, Durham, North Carolina, United States of America; University of Florida, United States of America

## Introduction

MicroRNAs (miRNAs) are an extensive family of small regulatory RNAs that function by binding to complementary mRNAs, primarily in the 3′ untranslated region (3′UTRs), and then inhibiting their expression by reducing mRNA translation and/or stability [1]. MiRNAs are initially transcribed as long pri-miRNAs, which are sequentially processed by the RNase III enzymes Drosha, in the nucleus, and Dicer, in the cytoplasm, to generate the mature, ∼22-nt miRNA [2]. This is then loaded into the RNA Induced Silencing Complex (RISC), which consists minimally of one of the four mammalian Argonaut proteins, Ago1 to Ago4, as well as a member of the GW182 protein family. MiRNAs function as guide RNAs to target RISC to complementary mRNA sequences on specific mRNA 3′UTRs. Analysis has revealed that complementarity to nucleotides 2 through 8 of the miRNA, the so-called seed region, is particularly important for effective RISC recruitment [1], although non-canonical sites, with incomplete seed complementarity, have also been reported [3]. Importantly, RISC recruitment to target sites that are occluded by RNA secondary structure or bound proteins is very inefficient [4].

## Viruses and MicroRNAs

Upon infection of a cell, viruses encounter a wide range of miRNA species, generally more than 50 different miRNAs per cell, and these miRNAs vary greatly between tissues. For example, miR-122 is expressed at very high levels in hepatocytes, but is absent from almost all other cells, while miR-1 is primarily expressed in muscle tissue and miR-128 in neuronal cells [5]–[7]. Indeed, many of the more than 1000 known human miRNA species show a tissue-specific expression pattern [8], meaning that viruses that infect multiple cell types need a way to avoid inhibition by a wide range of miRNAs with distinct mRNA-targeting specificities.

Analyses of the interactions of viruses with cellular miRNAs have revealed that viruses can influence cellular miRNA biogenesis and effector mechanisms in several different ways. Viruses can clearly benefit from miRNA expression. For example, almost all herpesviruses that have been examined express substantial numbers of miRNAs, and these can facilitate viral replication and/or regulate viral entry or exit from latency [9]. While some DNA viruses also express miRNAs, including adeno and polyoma viruses, miRNAs have not been detected in any RNA viruses examined so far, with the exception of the retrovirus bovine leukemia virus (BLV), which transcribes short, pol III-driven miRNA precursors from integrated BLV proviruses [10]. Viruses can also benefit from cellular miRNA species, with the clearest example being Hepatitis C virus (HCV), which requires miR-122 for replication [5]. Moreover, several other viruses have been reported to induce specific cellular miRNAs, and it has been demonstrated that, in some instances, this induction facilitates viral replication in culture, apparently by down-regulating specific cellular mRNA targets with antiviral potential [11], [12].

While certain viruses can clearly benefit from cellular miRNAs, it has been unclear how viruses avoid inhibition of viral mRNA function by cellular miRNAs. Indeed, several reports demonstrating inhibition of viruses by cellular miRNAs have been published [13]–[15]. However, especially given that cellular miRNAs are highly conserved during evolution [1], it seems unlikely that viruses would fail to evolve mechanisms to prevent cellular miRNA-mediated inhibition in their normal target tissues. What these might be, however, is currently unclear. Several possible mechanisms can be proposed:

1. **Viruses block miRNA function.** This appears rare, as virus-infected cells generally contain normal levels of miRNAs, and most viruses can be inhibited by specific small interfering RNAs (siRNAs), which function indistinguishably from miRNAs in mammalian cells [16], or by insertion of target sites for endogenous cellular miRNAs into viral transcripts [17]–[20]. Indeed, the use of inserted cellular miRNA target sites as a way of inhibiting viral replication in tissues that express the cognate endogenous miRNA, while allowing unhindered viral replication in cells that lack this miRNA, has considerable potential in facilitating the development of novel attenuated viral vaccines or in targeting oncolytic viral vectors away from normal tissues [17]–[20]. Uniquely, in the case of poxviruses, it has been shown that miRNAs are degraded in infected cells [21]. In contrast, HIV-1 and influenza viruses, despite early reports to the contrary, have now been clearly shown to not block miRNA function [19], [22], and indeed, the tissue and/or species tropism of influenza virus can be readily manipulated by insertion of target sites for endogenous miRNAs [19].
2. **Viruses evolve to avoid 3′UTR targets complementary to cellular miRNAs.** Because full complementarity to the seed is generally critical for miRNA inhibition, single nucleotide mutations should block inhibition [1]. However, for viruses that can replicate in several different tissues, each expressing more than 50 distinct miRNAs, complete avoidance of all miRNAs may be very difficult to achieve. Nevertheless, especially for viruses that display a narrow tissue tropism, this mechanism seems very likely to be important.
3. **Viruses evolve very short 3′UTRs.** RISC recruitment to open reading frames (ORFs) does not effectively inhibit mRNA function, most probably because translating ribosomes sweep bound RISCs off the mRNA [1]. Therefore, mRNAs with very short 3′UTRs would be expected to be relatively refractory to miRNA-mediated inhibition. In fact, many RNA viruses express mRNAs bearing short 3′UTRs, and the short 3′UTRs that are present are often highly structured, which is predicted to also inhibit RISC binding [4]. RNA virus families that appear likely to use this strategy to avoid inhibition by endogenous cellular miRNAs include flaviviruses, picornaviruses, rhabdoviruses, and reoviruses.
4. **Viruses evolve structured 3′UTRs.** Some viruses, especially retroviruses, alphaviruses, and coronaviruses, contain extensive 3′UTRs in at least some viral mRNA species. For example, the HIV-1 mRNA that encodes Gag and Gag-Pol has a 3′UTR that is several thousand nucleotides in length. Similarly, the coronavirus mRNA encoding the viral ORF1a and ORF1b proteins has a 3′UTR more than 10,000 nucleotides in length. How do these very long 3′UTRs avoid functioning as targets for multiple miRNAs? One possibility is that these 3′UTRs have evolved high levels of RNA secondary structure, which would be predicted to globally restrict binding by miRNA-programmed RISCs [4].

Relatively little is known about the secondary structure of viral RNAs, although some data suggest that high levels of secondary structure are a common feature [23]. One viral RNA that has been examined in detail is the HIV-1 RNA genome, which also functions as the mRNA for the viral Gag and Gag-Pol proteins. This RNA has been shown to fold into an extensive secondary structure with relatively few areas that are unfolded and hence, presumably, are available for RISC binding [24]. This prediction has been validated by a comprehensive analysis of the susceptibility of the HIV-1 genome to small interfering RNAs (siRNAs), which in mammalian cells function indistinguishably from miRNAs [16]. These researchers generated over 9,000 siRNAs specific for the HIV-1 genome by sliding the siRNA target along the viral genome by one-nucleotide increments [25]. Relatively few of these siRNAs were found to inhibit HIV-1 replication and gene expression effectively, and those that did were predicted to bind to the few regions of the viral RNA genome that, using biochemical approaches, were predicted to adopt an open, unfolded conformation [24], [25].

Recently, the ability of the HIV-1 genome to bind to endogenous cellular miRNAs in relevant target cells (CD4^+^ T cells) or in a non-physiological target cell (HeLa cells) has been examined using a technology called photo-activatable ribonucleoside-enhanced crosslinking and immunoprecipitation (PAR-CLIP). The PAR-CLIP technique involves pulsing cells with the highly photoactivatable uridine analog 4-thiouridine and then crosslinking endogenous RNAs to bound proteins by irradiation at 365 nm [26]. Crosslinked proteins, and the bound RNAs, are recovered by immunoprecipitation of RISC using an Ago2-specific monoclonal antibody and the binding site footprinted by RNase treatment. The RISC binding sites are then comprehensively identified by deep sequencing of these small RNAs to generate sequence clusters that can be aligned to endogenous miRNA species.

Analysis of RISC binding to the HIV-1 genome indeed identified several binding sites that were occupied by RISCs programmed by endogenous cellular miRNAs and some of these could be shown, by indicator assays, to confer a modest repression of mRNA function [27]. However, perhaps the more interesting finding was that viral mRNAs, despite contributing more than 10% of the total mRNA transcriptome in HIV-1 infected cells, in fact gave rise to only approximately 0.2% of all assignable RISC binding sites, with the remaining approximately 99.8% being contributed by cellular mRNAs. That is, viral mRNAs are, at a minimum, 50-fold less likely to bind RISC than are cellular mRNAs, consistent with the idea that HIV-1-encoded mRNAs, at least, have evolved to globally avoid cellular miRNAs by adopting RNA secondary structures that preclude RISC binding.

While a more complete understanding of the interaction of viruses with cellular miRNAs must await a more detailed dissection of the effect of endogenous miRNAs on a wide range of viral species, current data suggest that viruses have likely evolved a number of strategies to avoid inhibition by these ubiquitous cellular regulatory RNAs. Whether the perturbation of these avoidance strategies has the potential to lead to the development of reagents that are useful in disease prevention, such as novel forms of attenuated viral vaccines, remains to be determined.
